# *Toxoplasma gondii* Genotype Determines Tim-3 Expression Levels in Splenic and Circulatory T Cells in Mice

**DOI:** 10.3389/fmicb.2018.02967

**Published:** 2018-12-04

**Authors:** Yiwei Zhang, Ning Jiang, Ting Zhang, Dawei Wang, Ying Feng, Xiaoyu Sang, Na Yang, Qijun Chen

**Affiliations:** Key Laboratory of Zoonosis, College of Animal Science and Veterinary Medicine, Shenyang Agricultural University, Shenyang, China

**Keywords:** *Toxoplasma gondii*, Tim-3, infection, immune cells, cytokine

## Abstract

*Toxoplasma gondii* is an obligatory intracellular parasite that causes a common infection in many warm-blooded animals. During infection, the host’s immune system plays an important role in confining the dissemination of the parasites in the hosts. T cell immunoglobulin- and mucin domain–containing molecule 3 (Tim-3) has been characterized as an important regulator in cell-mediated immune responses in various infections. Here, we compared Tim-3 expression on splenic and circulatory T, B cells and a few cytokines in the sera of mice infected with the more virulent type I (RH) vs. the low virulent type II (ME49) strain. Tim-3 expression on the splenic and circulatory T cells of mice infected with *T. gondii* (RH strain) was higher than that in mice infected with *T. gondii* (ME49 strain). *T. gondii* infection reduced the proportion of splenic helper T cells (Th) and cytotoxic T cells (Tc) and increased Tim-3 expression. Further, serum levels of interleukin (IL)-2, interferon γ, tumor necrosis factor (TNF)-α, IL-12p70, IL-22, IL-17A, and IL-5 increased significantly after infection. Mice infected with *T. gondii* (ME49 strain) showed higher levels of TNF-α, IL-17A, IL-12p70, and IL-22 than that infected by the RH strain. Our study revealed that *T. gondii* strains may have their inherent ability in triggering different host immune responses, which may explain the clinical variation in diseases severity after infection.

## Introduction

The parasite *Toxoplasma gondii* can infect almost all warm-blooded animals, including humans (Webster, 2010). It can cause widespread subclinical human infection (Mccabe and Remington, 1988) and severe disease in immunocompromised patients, especially people infected with HIV (Luft and Remington, 1988). Previous study (Howe and Sibley, 1995) has indicated that *T. gondii* strains fall into three distinct clonal lineages: type I (e.g., RH and GT-1), type II [e.g., ME49 and its derivatives (PDS, PLK, PTg)], and type III (e.g., CEP, CTg, VEG). The genetic difference of these strains is around 1% (Su et al., 2003). Different *T. gondii* genotypes might differ in their capacity for inducing pathology or occurrence in a particular animal species (Jia et al., 2013). A subpopulation of a type I strain exhibited faster migration *in vitro* compared with type II and type III strains (Antonio and David, 2002). Further, type I strains transmigrated across mouse intestinal epithelium and penetrated the vascular endothelium more quickly *ex vivo* (Antonio and David, 2002). Type II, but not type I or type III strains, mainly dominated in patients with AIDS and congenital infections (Honoré et al., 2000). Infection with type II strain parasites, and not type I or type III strains, stimulated the production of proinflammatory cytokines, and in macrophages, particularly high levels of the T helper 1 cell (Th1)-polarizing cytokine, IL-12, were highly dependent on the parasite genotype (Robben et al., 2004).

In *T. gondii* primary infection, both CD4^+^ and CD8^+^ T cells of the host participate in the immune response to the parasite (Purner et al., 1996). CD8^+^ T cells may determine cyst numbers in *T. gondii*–infected murine models (Brown and Mcleod, 1990). CD4^+^ T cells can control the growth of tachyzoites, and upon appropriate stimulation, all CD4^+^ T cells will produce interleukin (IL)-2 and interferon (IFN)-γ, which are central to resistance to *T. gondii* infection (Canessa et al., 1988). B cell–deficient mice vaccinated with an attenuated strain of *Toxoplasma* (ts-4) do not survive after challenge with T. gondii (RH strain) tachyzoites (Sayles et al., 2000). However, the immunized mice are protected after oral challenge with a mildly virulent *T. gondii* strain (Johnson et al., 2004).

In the immune response to various parasite infections and disease models, T cell immunoglobulin- and mucin domain–containing molecule 3 (Tim-3) plays a widespread and complex role in both adaptive and innate immunity (Freeman et al., 2010). Tim-3 is mainly expressed on activated Th1 cells (Monney et al., 2002). Tim-3 is also expressed on non–T cells, including dendritic cells, monocytes, macrophages, natural killer (NK) and NK T cells, mast cells, and at lower levels in Th17 cells (Nakayama et al., 2009; Gleason et al., 2012; Ndhlovu et al., 2012). In *Plasmodium* infection, increased Tim-3 expression results in lymphocyte exhaustion, while Tim-3 signaling blockade restores lymphocyte activity (Hou et al., 2016); blocking Tim-3 also improves the phagocytosis and parasitic mediator production of murine splenic macrophages (Hou et al., 2017). Further, differential Tim-3 expression of immune cells from the spleen, mesenteric lymph nodes (Berrocal Almanza et al., 2013), and brain (Wu et al., 2014) has been observed during *T. gondii* infection in mice from different backgrounds. However, there has been no further investigation on Tim-3 expression on the immune cells of hosts infected with different *T. gondii* genotypes.

In the present study, we investigated Tim-3 expression and its role in regulating cytokine production in mice infected with different T. gondii genotypes. The data indicates that differences in host’s Tim-3 expression and cytokine production triggered by T. gondii may contribute to the virulent characteristics of these parasite strains.

## Materials and Methods

### Ethics Statement

Female BALB/c mice (6–8 weeks old) were maintained in our animal facility under specific pathogen–free conditions. All animal procedures performed in the present study were conducted following the animal husbandry guidelines of Shenyang Agricultural University; the Ethical Committee of Shenyang Agricultural University approved the laboratory animal experiments (Permit No. SYXK < Liao > 2011-0001).

### Infection Experiments

The tachyzoites of the T. gondii (RH strain) were obtained from the peritoneal fluid of BALB/c mice that had previously been inoculated with the parasite strain. The parasites of ME49 strain were, in first step, proliferated in monkey kidney adherent fibroblasts (Vero cells) at 37°C in a 5% CO2 incubator. Then nearly 108 parasites of ME49 strain were injected into the peritoneal cavity of mice. The peritoneal fluid of mice was repeatedly squeezed with a 5 ml curved syringe, then parasites were filtered through a 5.0 μm filter and checked under the microscope to ensure that they are all single bradyzoites. For the infection assays, mice (*n* = 20 per group) were each infected with 100 ME49 bradyzoites (ME49 group) or 100 RH tachyzoites (RH group) in 0.2 ml 1 × phosphate-buffered saline (pH 7.4).

### Preparation of Splenic and Circulatory Immune Cells

The spleens of infected mice and healthy mice (Control group) were cut into pieces, minced, and pressed into a 70-μm cell strainer. Red blood cells (RBCs) were depleted with RBC lysis solution (155 mM NH_4_Cl, 10 mM KHCO_3_, 0.11 mM EDTA Na_2_, pH 7.2). Anticoagulated blood was obtained and the circulatory immune cells were isolated by depleting RBCs as described above. The mouse (*n* = 5 per group) splenic immune cells and circulatory immune cells were collected at day 3, 5, 7, and 9 post-infection (Supplementary Figure S1).

### Flow Cytometry

Cells were stained with Zombie NIR Fixable Viability dye (catalog no. 423106; BioLegend, San Diego, CA, United States), which is non-permeant to live cells but is permeant to cells with compromised membranes, i.e., dead cells. The cells were then pre-incubated with purified anti-mouse CD16/32 antibody (catalog no. 101310; BioLegend) to block non-specific immunoglobulin binding to the Fc receptors. Finally, the cells were incubated with specific antibodies or isotype controls according to the manufacturers’ guidelines. The antibodies used were: Pacific Blue anti-mouse CD45; allophycocyanin-conjugated (APC) anti-mouse CD3; fluorescein isothiocyanate–conjugated (FITC) anti-mouse CD8a; peridinin chlorophyll protein complex–conjugated (PerCP) anti-mouse CD4; FITC anti-mouse CD19; phycoerythrin-conjugated (PE) anti-mouse CD366; Pacific Blue Rat immunoglobulin (Ig)G2b, κ Isotype Ctrl; APC Rat IgG2b, κ Isotype Ctrl; FITC Rat IgG2a, κ Isotype Ctrl; PerCP Rat IgG2b, κ Isotype Ctrl; and PE Rat IgG2a, κ Isotype Ctrl (all from BioLegend). The cells were detected and analyzed using a FACSAria III flow cytometer (BD Biosciences, San Jose, CA, United States); positive cells were defined using an isotype control or a fluorescence minus one control.

### Cytokine Detection

IL-2, IL-4, IL-17A, tumor necrosis factor (TNF)-α, IL-5, IL-22, and IFN-γ levels in the mouse serum were determined using a LEGENDplex Mouse Th Cytokine Panel (catalog no. 740005; BioLegend). All serum samples were diluted 1:1 in assay buffer. Samples were treated according to the BioLegend standard protocol and were examined using FACSAria III (BD Biosciences) driven by FACSDiva software (BD Biosciences). IL-12p70 was assayed by ELISA in microplates (catalog no. 1211202; DAKEWE BIOTECH CO., LTD.) according to the standard protocol.

### Statistical Analysis

All data were analyzed with GraphPad Prism 5.0 (GraphPad Software, Inc., La Jolla, CA, United States) and PASW Statistics 18 (IBM Co., Armonk, NY, United States). The results were analyzed using a two-tailed paired *t*-test. The mean and standard deviation (SD) were determined using 3–5 biological replicates. *P* < 0.05 was considered significant. Cytokine calculations were performed using the LEGENDplex 8.0 application (VigeneTech Inc., Carlisle, MA, United States) and ELISACalc data program. The heatmap was drawn using Heml 1.0.3.7 software (Huazhong University of Science and Technology, Hubei, Wuhan, China).

## Results

### The Splenic T and B Cells of *T. gondii* (RH Strain)–Infected Mice Showed Higher Tim-3 Expression Than That of Mice Infected With *T. gondii* (ME49 Strain)

**FIGURE 1:**
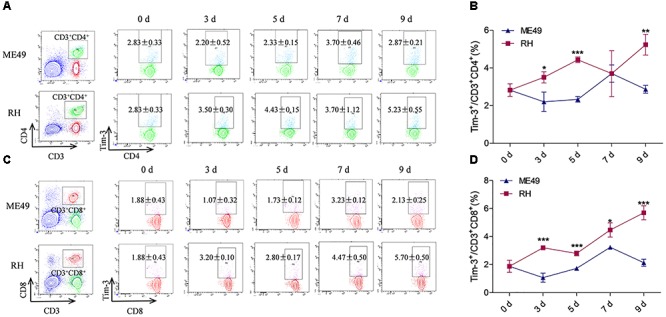
Tim-3 expression on splenic T cells in mice infected with T. gondii of the RH or ME49 strain. Tim-3 expression on splenic Th cells **(A,B)** and Tc cells **(C,D)** was detected by flow cytometry at day 3, 5, 7, and 9 post-infection. (**A**, left panel) The gating strategy of Th cells. (**A**, right panel) Representative dot plots of Tim-3 expression on CD3^+^CD4^+^ Th cells. **(B)** Comparisons of the proportions of Tim-3^+^ cells within the CD3^+^CD4^+^ cell population between the ME49 and RH groups. (**C**, left panel) The gating strategy of Tc cells. (**C**, right panel) Representative dot plots of Tim-3 expression on CD3+CD8+ Tc cells. **(D)** Comparisons of the proportions of Tim-3^+^ cells within the CD3+CD8+ cell population between ME49 group and RH group The results are representative of three independent experiments with 3–5 mice per group per experiment; data are the means ± SDs. ^∗^*P* < 0.05, ^∗∗^*P* < 0.01, ^∗∗∗^*P* < 0.001; ^∗^indicates comparisons to the ME49 group.

Tim-3^+^ cells in the spleen and mesenteric lymph nodes are increased after *T. gondii* infection (Berrocal Almanza et al., 2013). Here, we investigated Tim-3 expression on splenic lymphocyte subpopulations in mice infected with different *T. gondii* genotypes. The splenic helper T (Th) cells and cytotoxic T (Tc) cells in the RH group had higher Tim-3 expression as compared to the ME49 group at most of the time points (Figures 1A–D). Although flow cytometry revealed nearly equal Tim-3 expression on the splenic CD3^+^CD4^+^ Th cells from the RH and ME49 groups at day 7, splenic Th cells in the RH group had higher Tim-3 expression at most of the time points compared to that of the ME49 group (Figures 1A,B, RH group vs. ME49 group: day 3, 3.50 ± 0.30 vs. 2.20 ± 0.52, *P* < 0.05; day 5, 4.43 ± 0.15 vs. 2.33 ± 0.15, *P* < 0.001; day 7, 3.70 ± 1.22 vs. 3.70 ± 0.46, *P* > 0.05; day 9, 5.23 ± 0.55 vs. 2.87 ± 0.21, *P* < 0.01).

Splenic CD3^+^CD8^+^ Tc cells from the RH group had higher Tim-3 expression compared to that of the ME49 group post-infection (Figures 1C,D, RH group vs. ME49 group: day 3, 3.20 ± 0.10 vs. 1.07 ± 0.32, *P* < 0.001; day 5, 2.80 ± 0.17 vs. 1.73 ± 0.12, *P* < 0.001; day 7, 4.47 ± 0.50 vs. 3.23 ± 0.12, *P* < 0.05; day 9, 5.70 ± 0.50 vs. 2.13 ± 0.25, *P* < 0.001).

Tim-3 expression on splenic B cells after infection with different *T. gondii* genotypes was also observed: the RH group had significantly higher expression than the ME49 group at day 3, 5, and 7 post-infection (Supplementary Figure S2). Although Tim-3 expression on the splenic B cells in the RH group remained higher than that of the ME49 group at day 9, it was not significant (Supplementary Figure S2 and data not shown).

### The Circulatory T Cells in *T. gondii* (RH Strain)–Infected Mice Had Higher Tim-3 Expression Than That of Mice Infected *T. gondii* (ME49 Strain)

**FIGURE 2:**
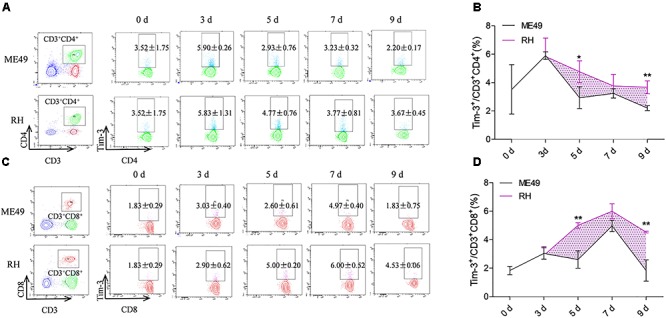
Tim-3 expression on circulatory T cells in mice infected with T. gondii of the RH or ME49 strain. Tim-3 expression on circulatory Th cells **(A,B)** and Tc cells **(C,D)** was detected by flow cytometry at day 3, 5, 7, and 9 post-infection. (**A**, left panel) The gating strategy of Th cells. (**A**, right panel) Representative dot plots of Tim-3 expression on CD3^+^CD4^+^ Th cells. **(B)** Comparisons of the proportions of Tim-3^+^ cells within the CD3^+^CD4^+^ cell population between ME49 group and RH group. (**C**, left panel) The gating strategy of Tc cells. (**C**, right panel) Representative dot plots of Tim-3 expression on CD3^+^CD8^+^ Tc cells. **(D)** Comparisons of the proportions of Tim-3^+^ cells within the CD3^+^CD8^+^ cell population between ME49 group and RH group. The results are representative of three independent experiments with 3–5 mice per group per experiment; data are the means ± SDs. ^∗^*P* < 0.05, ^∗∗^*P* < 0.01; ^∗^indicates comparisons to the ME49 group.

Circulatory Th cells and Tc cells in the RH group had higher Tim-3 expression compared to that of the ME49 group at most of the time points (Figures 2A–D). The splenic Th cells in the RH group had almost identical Tim-3 expression nearly equal to that of the ME49 group at day 3 post-infection (Figures 2A,B, RH group vs. ME49 group: 5.83 ± 1.31 vs. 5.90 ± 0.2, *P* > 0.05). The circulatory Th cells in the RH group had significantly higher Tim-3 expression compared to that of the ME49 group at day 5 and 9 post-infection (Figures 2A,B, RH group vs. ME49 group: day 5, 4.77 ± 0.76 vs. 2.93 ± 0.76, *P <* 0.05; day 9, 3.67 ± 0.45 vs. 2.20 ± 0.17, *P* < 0.01). Although Tim-3 expression on splenic Th cells in the RH group remained higher than that of the ME49 group at day 7 post-infection, it was not significant (Figures 2A,B, RH group vs. ME49 group: 3.77 ± 0.81 vs. 3.23 ± 0.32, *P* > 0.05).

Tim-3 expression on splenic Tc cells in the RH group was almost identical to that of the ME49 group at day 3 post-infection (Figures 2C,D, RH group vs. ME49 group: 2.90 ± 0.62 vs. 3.03 ± 0.40, *P* > 0.05). The circulatory Tc cells in the RH group had significantly higher Tim-3 expression compared to that of the ME49 group at day 5 and 9 post-infection (Figures 2C,D, RH group vs. ME49 group: day 5, 5.00 ± 0.20 vs. 2.60 ± 0.61, *P* < 0.01; day 9, 4.53 ± 0.06 vs. 1.83 ± 0.75, *P* < 0.01). Although Tim-3 expression on the splenic Tc cells in the RH group remained higher than that of the ME49 group at day 7 post-infection, it was not significant (Figures 2C,D, RH group vs. ME49 group: 6.00 ± 0.52 vs. 4.97 ± 0.40, *P* > 0.05).

### *T. gondii* Infection Induced Higher Tim-3 Expression and Reduced Proportion of Splenic T Lymphocytes

**FIGURE 3:**
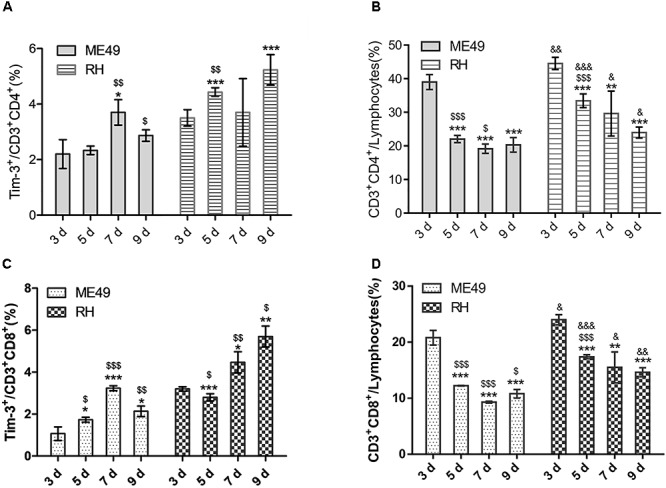
*T. gondii* infection reduced splenic T lymphocytes and increased Tim-3 expression *in vivo*. **(A)** Histograms showing the frequency of Tim-3^+^ cells in CD3^+^CD4^+^ T cells at day 3, 5, 7, and 9 post-infection. **(B)** Histograms showing the proportions of CD3^+^CD4^+^ Th cells in splenic lymphocytes at day 3, 5, 7, and 9 post-infection. **(C)** Histograms showing the frequency of Tim-3^+^ cells in CD3^+^CD8^+^ T cells at day 3, 5, 7, and 9 post-infection. **(D)** Histograms showing the proportions of CD3^+^CD8^+^ Tc cells in splenic lymphocytes at day 3, 5, 7, and 9 post-infection. The results are representative of three independent experiments with 3–5 mice per group per experiment; data are the means ± SDs. ^∗,$,&^*P* < 0.05, ^∗∗,$$,&&^*P* < 0.01, ^∗∗∗,$$$,&&&^*P* < 0.001; ^∗^indicates comparisons to day 3, ^$^indicates comparisons to the previous time point. ^&^indicates RH group comparisons to the ME49 group.

We observed increased Tim-3 expression on splenic CD3^+^CD4^+^ Th cells (Figure 3A, left) accompanied by a reduction in CD3^+^CD4^+^ Th cells (Figure 3B, left) from day 3–7 post-infection in the ME49 group. Although Tim-3 expression on CD3^+^CD4^+^ Th cells decreased at day 9 post-infection, it remained higher (but not significantly) than that on day 3 post-infection (Figure 3A, left, day 3 vs. day 9: 2.20 ± 0.52 vs. 2.87 ± 0.21, *P* > 0.05), and the proportion of splenic CD3^+^CD4^+^ Th cells at day 9 post-infection was significantly lower than that on day 3 post-infection (Figure 3B, left, day 3 vs. day 9: 39.00 ± 2.21 vs. 20.30 ± 2.16, *P* < 0.001) in the ME49 group. In the RH group, there was an increase in Tim-3 expression on the splenic CD3^+^CD4^+^ Th cells (Figure 3A, right) accompanied by a reduction of CD3^+^CD4^+^ Th cells (Figure 3B, right) on day 3–9 post-infection.

Further, there was increased Tim-3 expression on the splenic CD3^+^CD8^+^ Tc cells (Figure 3C, left) accompanied by a reduction of CD3^+^CD8^+^ Tc cells (Figure 3D, left) on day 3–7 post-infection in the ME49 group. Moreover, Tim-3 expression on CD3^+^CD8^+^ Tc cells decreased at day 9 post-infection, but remained higher than that on day 3 post-infection (Figure 3C, left, day 3 vs. day 9: 1.07 ± 0.32 vs. 2.13 ± 0.25, *P* < 0.05), and the proportion of splenic CD3^+^CD8^+^ Tc cells at day 9 post-infection remained lower than that on day 3 post-infection (Figure 3D, left, day 3 vs. day 9: 20.80 ± 1.31 vs. 10.80 ± 0.75, *P* < 0.05) in the ME49 group. In the RH group, there was increased Tim-3 expression on the splenic CD3^+^CD8^+^ Tc cells (Figure 3C, right) accompanied by a reduction of CD3^+^CD8^+^ Tc cells (Figure 3D, right) at day 7 and 9 post-infection.

**FIGURE 4:**
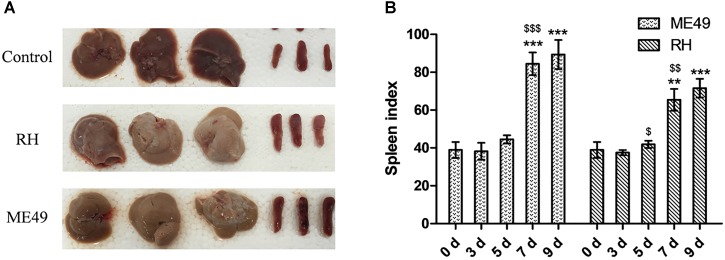
Splenomegaly post-infection. The spleen and liver were observed 7 days post-infection, and the spleen indexes [spleen weight (mg) × 10/body weight (g) × 100%] were calculated. **(A)** Representative images of spleen and liver from the Control, RH, and ME49 groups. **(B)** Spleen index of the ME49 and RH groups. The results are representative of three independent experiments with 3–5 mice per group per experiment; data are the means ± SDs. ^$^*P* < 0.05, ^∗∗,$$^*P* < 0.01, ^∗∗∗,$$$^*P* < 0.001; ^∗^indicates comparisons to day 0, ^$^indicates comparisons to the previous time point.

Splenomegaly was observed post-infection (Figure 4A). The spleen indexes of the ME49 and RH groups both increased at day 7 and 9 post-infection (Figure 4B). Further, the ME49 group had a higher spleen index than the RH group at day 7 and 9 post-infection (Figure 4B, ME49 group vs. RH group: day 7, 84.48 ± 5.99 vs. 65.48 ± 5.75, *P* < 0.05; day 9, 89.37 ± 7.65 vs. 71.60 ± 4.98, *P* < 0.05).

### Serum Proinflammatory Cytokines Increased After Infection and the Cytokine Levels Was Associated With *T. gondii* Genotype

**FIGURE 5:**
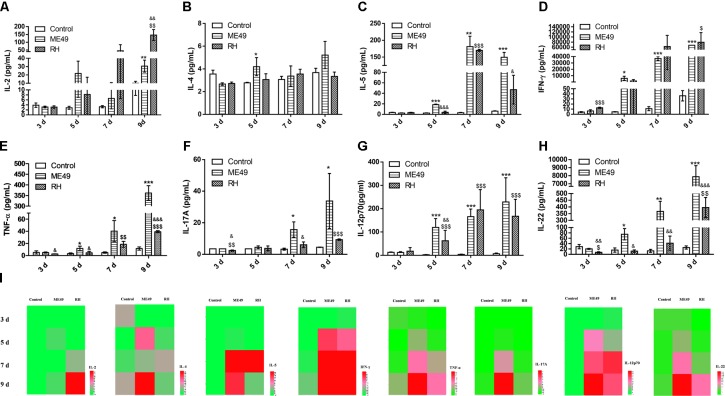
Cytokine production during infection with T. gondii of the RH or ME49 strain. **(A–H)** Comparisons of cytokine levels (IL-2, IL-4, IL-5, IFN-γ, TNF-α, IL-17A, IL-12p70, and IL-22) in sera from Control, ME49, and RH groups. **(I)** Heatmaps directly showing the difference in the cytokines detected. The results are representative of three independent experiments with 3–5 mice per group per experiment; data are the means ± SDs. ^∗,$,&^*P* < 0.05, ^∗∗,$$,&&^*P* < 0.01, ^∗∗∗,$$$,&&&^*P* < 0.001; ^∗^indicates ME49 group comparisons to the control group, ^$^indicates RH group comparisons to the control group, ^&^indicates RH group comparisons to the ME49 group.

Cytokines play significant roles in *T. gondii*–resistant hosts (Mahmoudi et al., 2017). To investigate lymphocyte activity after infection, we quantitatively determined the presence of IL-2, **IFN-γ**, TNF-α, IL-4, IL-5, IL-17A, IL-12p70, and IL-22 (Figures 5A–H) in mouse sera at day 3, 5, 7, and 9 post-infection; the sera of healthy mice were used as controls. Type I cytokines such as IL-2 (Figures 5A,I), IFN-γ (Figures 5D,I), and TNF-α (Figures 5E,I) increased gradually in the sera of both the RH and ME49 groups, and there was an earlier significant increase of IFN-γ at day 3 post-infection in the RH group (Figure 5D, RH group vs. Control group: 12.74 ± 1.15 vs. 4.33 ± 0.88, *P* < 0.05). At day 9 post-infection, the RH group had higher IL-2 levels than the ME49 group (Figure 5A). However, the RH group had lower TNF-α levels than the ME49 group at day 3, 5, and 9 post-infection (Figure 5E). There were no significant changes in the secretion of the type II cytokine IL-4 (Figures 5B,I), but type II cytokine IL-5 (Figures 5C,I) was increased post-infection; the RH group had lower IL-5 levels than the ME49 group at day 5 and 9 post-infection (Figures 5C,I). There was a significant increase in the type 17 cytokines IL-17A (Figures 5F,I) and IL-22 (Figures 5H,I) in both the RH and ME49 group. The RH group had lower IL-17A levels than the ME49 group at day 3 and 7 post-infection (Figure 5F). An increase secretion of IL-12 was determined post infection and the production of IL-12 was significantly lower in RH group than ME49 group at day 5 (Figure 5G). The RH group had lower IL-22 levels than the ME49 group at day 3, 5, 7, and 9 post-infection (Figure 5H).

### Mice Infected With *T. gondii* (RH Strain) Had More Proportion of Splenic Th, Splenic Tc, Circulatory Tc Cells and Less Proportion of Splenic B Cells Than That Infected With *T. gondii* (ME49 Strain)

We compared the frequency of T and B cells in the splenic and circulatory lymphocytes of mice infected with different *T. gondii* genotypes. Mice infected with RH tachyzoites had weaker splenic B cell response at day 3 and 5 post-infection; at day 7 and 9 post-infection, the frequency of splenic B cells in the RH group remained lower than that of the ME49 group, but it was not significant (Supplementary Figure S3, RH group vs. ME49 group: day 3, 21.38 ± 4.00 vs. 40.44 ± 4.07, *P* < 0.001; day 5, 33.52 ± 2.66 vs. 52.44 ± 4.28, *P* < 0.001; day 7, 45.38 ± 12.58 vs. 57.28 ± 2.01, *P* > 0.05; day 9, 51.74 ± 4.32 vs. 58.50 ± 5.94, *P* > 0.05). The RH group had significantly more population of splenic Th and Tc cells than the ME49 group at day 3, 5, 7, and 9 post-infection (Figures 4B,D).

**FIGURE 6:**
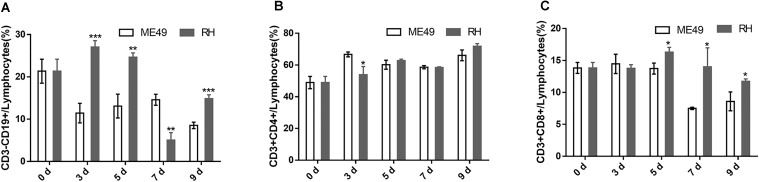
The proportion of circulatory T and B cells in mice after infection with *T. gondii* of the RH or ME49 strain. Circulatory immune cells **(A–C)** were collected at day 0, 3, 5, 7, and 9 post-infection. **(A)** The proportions of CD3^-^CD19^+^ B cells in circulatory lymphocytes. **(B)** The proportions of CD3^+^CD4^+^ Th cells in circulatory lymphocytes. **(C)** The proportions of CD3^+^CD8^+^ Tc cells in circulatory lymphocytes. The results are representative of three independent experiments with 3–5 mice per group per experiment; data are the means ± SDs. ^∗^*P* < 0.05, ^∗∗^*P* < 0.01, ^∗∗∗^*P* < 0.001; ^∗^indicates comparisons to the ME49 group.

The RH group had significantly more proportion of circulatory B cells than the ME49 group at day 3, 5, and 9 post-infection, but had lower frequency of circulatory B cells at day 7 post-infection (Figure 6A, RH group vs. ME49 group: day 3, 27.07 ± 1.50 vs. 11.43 ± 2.32, *P* < 0.001; day 5, 24.67 ± 1.01 vs. 13.10 ± 2.81, *P* < 0.01; day 7, 5.07 ± 1.75 vs. 14.60 ± 1.30, *P* < 0.01; day 9, 14.87 ± 0.91 vs. 8.53 ± 0.76, *P* < 0.001). There was no significant difference in the population of circulatory Th cells (Figure 6B). The RH group had more proportion of circulatory Tc cells at day 5, 7, and 9 post-infection than the ME49 group (Figure 6C, RH group vs. ME49 group: day 5, 16.30 ± 0.75 vs. 13.73 ± 0.85, *P* < 0.05; day 7, 14.00 ± 2.98 vs. 7.50 ± 0.17, *P* < 0.05; day 9, 11.73 ± 0.38 vs. 8.60 ± 1.47, *P* < 0.05).

## Discussion

A type I membrane protein, Tim-3 is an important immune regulator that plays widespread and complex roles in the immune system. Tim-3 induces T cell apoptosis through interaction with galectin-9 (Tang and Lotze, 2012). Several studies have shown that Tim-3 can suppress certain aspects of cell function, including enforcing CD8^+^ T cell exhaustion (Fourcade et al., 2010), suppressing IL-12 secretion (Tomkowicz et al., 2015). During *Plasmodium* infection, blocking of Tim-3 signaling with an anti–Tim-3 antibody restored lymphocyte activity (Hou et al., 2016) and improves phagocytosis and parasitic mediator production of murine splenic macrophages (Hou et al., 2017). In *T. gondii* infection, both susceptible C57BL/6 and resistant BALB/c mice had increased frequencies of Tim-3^+^ cells in the spleen and mesenteric lymph nodes (Berrocal Almanza et al., 2013). Further, differential Tim-3 expression by immune cells from the spleen, mesenteric lymph nodes (Berrocal Almanza et al., 2013), and brain (Wu et al., 2014) has been observed in mice from different backgrounds during *T. gondii* infection. However, there has been no further investigation on Tim-3 expression on the immune cells of hosts infected with different *T. gondii* genotypes. Our results revealed the expression of Tim-3 on splenic and circulatory Th and Tc cells in mice was closely associated to the parasite pathogenicity, virulent parasites induced higher Tim-3 expression on splenic and circulatory T cells.

In natural oral infections, *T. gondii* initially crosses the intestinal epithelium, disseminates into the deep tissues, and traverses biological barriers to reach immunologically privileged sites. In protozoan pathogens, growth rate is a common virulence trait, and *T. gondii* virulent often correlates with its growth rate (Kaufman et al., 1959). In mice, the genotype influences strain pathogenicity: virulent type I strains consistently display greater migratory capacity compared to the nonvirulent type II and type III strains (Antonio and David, 2002). Tim-3 is often correlated with apoptosis (Fourcade et al., 2010), and virulent strains have superior migratory and invasion capacity (Antonio and David, 2002), which may lead to earlier and faster host cell lysis. Here, Tim-3 expression on critical populations of splenic and circulatory Th cells and Tc cells in mice infected with type I RH *T. gondii*, a virulent strain, was higher, and *T. gondii* infection reduced splenic T lymphocytes and increased Tim-3 expression *in vivo*.

Spleen enlargement may be the first sign of a serious disorder (Motyckova and Steensma, 2012). Monoclonal proliferation from malignant transformation of a lymphoid cell or polyclonal lymphocyte proliferation may enlarge the spleen. Spleen enlargement can also occur due to infiltration of the spleen by nonlymphoid cells, such as inflammatory reaction by neutrophils (Motyckova and Steensma, 2012). Here, we observed post-infection splenomegaly. Study has also demonstrated a direct correlation between the degree of splenomegaly and IgM level produced by B cells (Wells, 1968, 1970). Compared to the RH group, the ME49 group had a higher spleen index and a higher population of splenic B cells which suggests that host infected with low virulent *T. gondii* strain has a higher chance to increase B cell populations for better protection against the parasite infection.

After *T. gondii* infection, overstimulation of the immune system will lead to high levels of Th1 cytokines, increased apoptosis, and organ damage (Gavrilescu and Denkers, 2001; Mordue et al., 2001). IL-12 is a proinflammatory cytokine that drives Th1 cell differentiation and forms a link between innate and adaptive immunity, IL-12 also stimulates IFN-γ production (Trinchieri, 2003). The combined effects of IL-12 and IFN-γ are essential to resistance to *T. gondii* and eventually provoke a very strong Th1-biased CD4^+^ and CD8^+^ T cell–mediated immune response (Miller et al., 2009). In the present study, IL-12 in the mouse sera increased after infection. This result further emphasize that *T. gondii* will trigger a strong Th1 immune response. While the production of IL-12 was significantly lower in the group infected with the RH strain than that infected with the ME49 strain at day 5. IFN-γ release assays can be used as early detection methods of *T. gondii* infection (Mahmoudi et al., 2017). Here, the RH group had significantly increased IFN-γ secretion at day 3 post-infection. TNF-α is also important for controlling resistance to acute (Johnson, 1992) and chronic (Deckertschlüter et al., 1998) *T. gondii* infections. Following stimulation with ME49 or RH strain tachyzoite lysate antigens, the spleen cells of mice infected with *T. gondii* (ME49 strain) had increased IL-4 production (Rodgers et al., 2005). Here, serum IL-4 which is produced by type 2 immune cells was neither increased nor decreased, IL-4 secreted by the spleen cells was not detected in this study. A previous study showed that after infection with *Babesia microti*, which is also an apicomplexan parasite, Th1 cell–mediated immunity is equally important in clearance of this intracellular pathogen. Further, the secretion of serum cytokines such as IL-4 was not significantly changed (Djokic et al., 2018). Our data further explained the previous findings at molecular level.

Conclusively, our results indicated that Tim-3 expression on splenic and circulatory T Cells and various cytokine responses in infected mice was closely associated with the *T. gondii* virulence.

## Author Contributions

The study was designed by QC. Experiments were performed by YZ, NJ, and TZ. Data were analyzed by QC, NJ, YZ, TZ, DW, YF, XS, and NY. Manuscript was written by QC, NJ, and YZ.

## Conflict of Interest Statement

The authors declare that the research was conducted in the absence of any commercial or financial relationships that could be construed as a potential conflict of interest.
