# Supplementary material for: Toxoplasma gondii Genotype Determines Tim-3 Expression Levels in Splenic and Circulatory T Cells in Mice
Source: Front Microbiol. 2018 Dec 4;9:2967. doi: 10.3389/fmicb.2018.02967 (PMC6288189; doi:10.3389/fmicb.2018.02967)
Supplement: Supplementary file 1 [file Data_Sheet_1.docx]

Supplementary Material

***Toxoplasma gondii* Genotype Determines Tim-3 Expression Levels in Splenic and Circulatory T Cells in Mice**

**Yiwei Zhang^†^, Ning Jiang^†^, Ting Zhang^†^, Dawei Wang, Ying Feng, Yao Wang, Ran Chen, Xiaoyu Sang, Na Yang, Qijun Chen^*^**

**^*^Correspondence:** Professor Qijun Chen: [qijunchen759@syau.edu.cn](mailto:qijunchen759@syau.edu.cn)

## Supplementary Figures


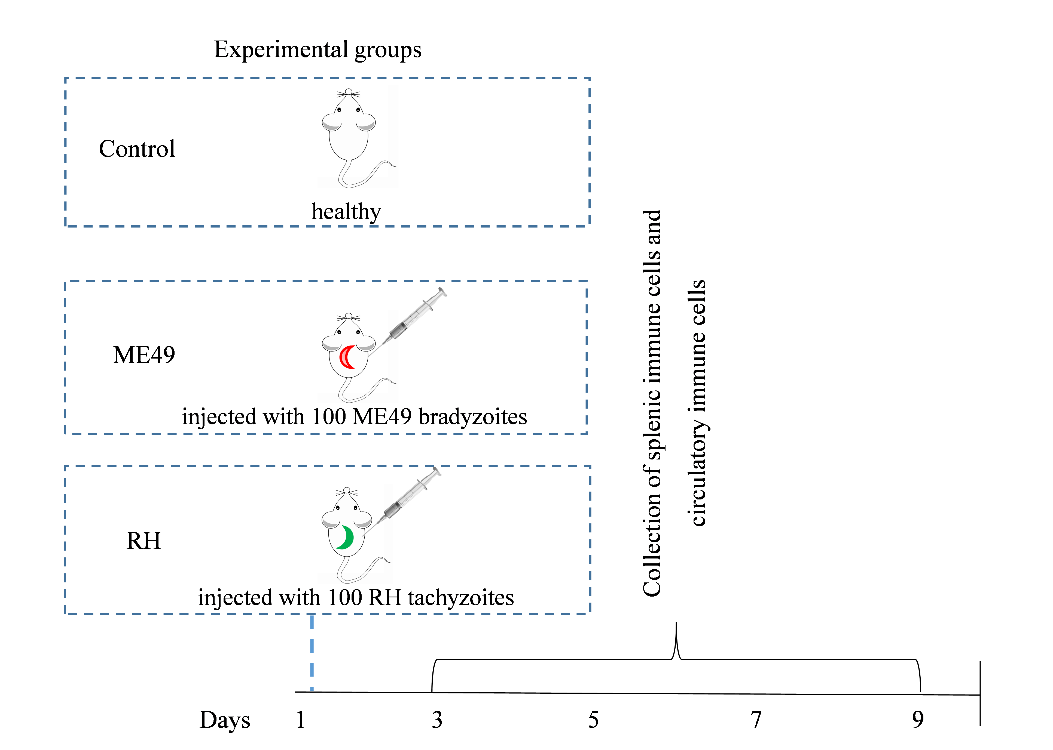


**SUPPLEMENTARY FIGURE S1.** Time points of splenic immune cell and circulatory immune cell collection. Sixty female BALB/c mice (20–25 g, 6–8 weeks old) were randomly divided into the Control group, ME49 group, and RH group (n = 20 per group). The ME49 and RH groups were peritoneally infected with 100 ME49 **bradyzoites** or 100 RH tachyzoites per mouse, respectively. Splenic immune cells and circulatory immune cells were collected at day 3, 5, 7, and 9 post-infection.


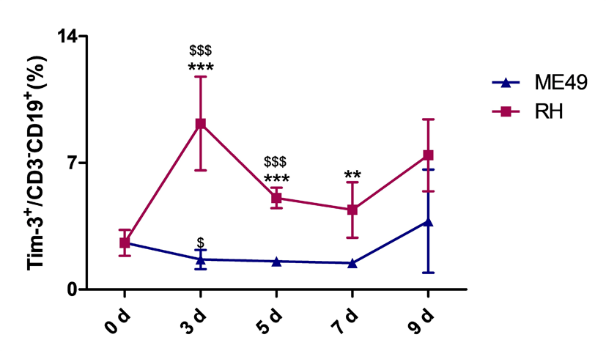


**SUPPLEMENTARY FIGURE S2.** Tim-3 expression on splenic B cells in mice infected with *T. gondii* strain RH and ME49. CD3^-^CD19^+^ B cells were collected at day 3, 5, 7, and 9 post-infection, Tim-3 expression on the cells was detected by flow cytometry. The results are representative of three independent experiments with 3–5 mice per group per experiment; data are the means ± SDs. ^$^ *P* < 0.05, ** *P* < 0.01, ***^, $$$^ *P* < 0.001; * indicates comparisons to day 0, ^$^ indicates comparisons to the previous time point.


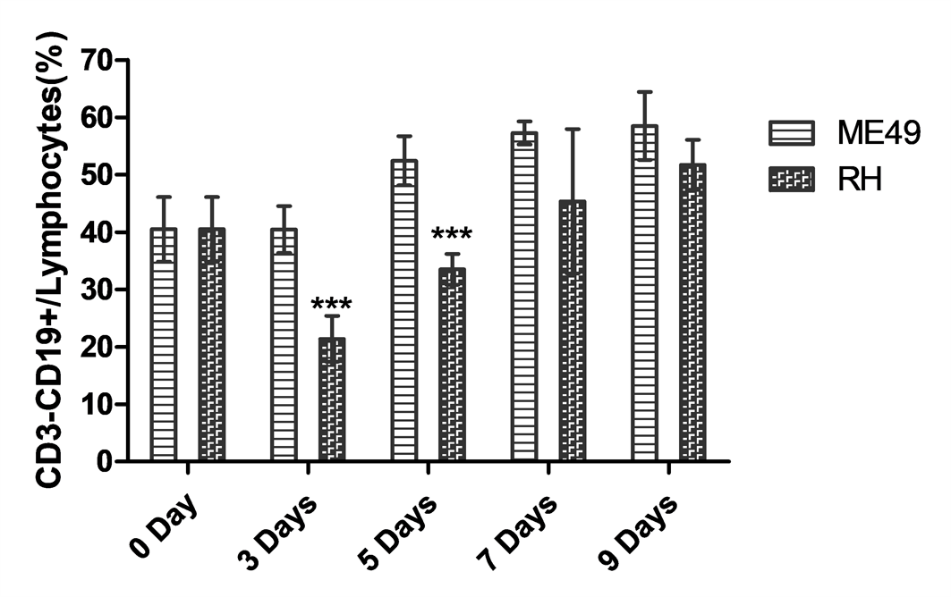


**SUPPLEMENTARY FIGURE S3.** The proportion of splenic B cells in mice after infection with *T. gondii* of the RH or ME49 strain. The results are representative of three independent experiments with 3–5 mice per group per experiment; data are the means ± SDs. **P* < 0.05, ***P* < 0.01, ****P* < 0.001; * indicates comparisons to the ME49 group.
